# Differential Repression of Alternative Transcripts: A Screen for miRNA Targets

**DOI:** 10.1371/journal.pcbi.0020043

**Published:** 2006-05-12

**Authors:** Matthieu Legendre, William Ritchie, Fabrice Lopez, Daniel Gautheret

**Affiliations:** INSERM ERM 206, Université de la Méditerranée, Marseille, France; University of Medicine and Dentistry of New Jersey, United States of America

## Abstract

Alternative polyadenylation sites produce transcript isoforms with 3′ untranslated regions (UTRs) of different lengths. If a microRNA (miRNA) target is present in the UTR, then only those target-containing isoforms should be sensitive to control by a cognate miRNA. We carried out a systematic examination of 3′ UTRs containing multiple poly(A) sites and putative miRNA targets. Based on expressed sequence tag (EST) counts and EST library information, we observed that levels of isoforms containing targets for miR-1 or miR-124, two miRNAs causing downregulation of transcript levels, were reduced in tissues expressing the corresponding miRNA. This analysis was repeated for all conserved 7-mers in 3′ UTRs, resulting in a selection of 312 motifs. We show that this set is significantly enriched in known miRNA targets and mRNA-destabilizing elements, which validates our initial hypothesis. We scanned the human genome for possible cognate miRNAs and identified phylogenetically conserved precursors matching our motifs. This analysis can help identify target-miRNA couples that went undetected in previous screens, but it may also reveal targets for other types of regulatory factors.

## Synopsis

MicroRNAs (miRNAs) are short RNA molecules that recognize specific target sequences in the 3′ region of mRNAs. These miRNAs can then specifically keep the mRNAs from being expressed, or translated into proteins. In this article, the authors ask what happens when a targeted mRNA has several forms differing by their 3′ regions. Such 3′ variations are very common. If two or more variations are present in a single mRNA, the result is two or more mRNAs with 3′ ends of different lengths. If an miRNA target is located between the two sites of variability, the shorter transcript should be target free and should escape miRNA-mediated inhibition, while longer transcripts should be inhibited. To test this hypothesis, the authors looked at mRNAs that had these variable 3′ ends. Variants containing targets for certain miRNAs appeared to be specifically underrepresented in tissues where these particular miRNAs are found. This principle was used to find other sequence patterns in 3′ regions that had a similar effect, and a list of 312 significant patterns was obtained. The authors then scanned genome sequences and identified possible cognate miRNAs for these patterns. This new knowledge will help further an understanding of how genes are controlled.

## Introduction

The current model of animal microRNA (miRNA) function posits that part of the 21–22nt miRNA sequence binds to the 3′ untranslated region (UTR) of a target mRNA, causing a downregulation of gene expression [1]. Target recognition most often involves a short 6–8 nt “seed” fragment at the miRNA 5′ end pairing to an exact complementary sequence in the 3′ UTR [2,3]. A typical animal miRNA may target in the order of 100 different genes [4,5]. Most animal miRNAs were believed to act by repressing translation, rather than by mRNA cleavage as observed in plants [6]. However, recent experimental evidence [5,7,8] is challenging this view. By introducing tissue-specific miR-1 and miR-124 miRNAs into HeLa cells, Lim et al. showed that (1) miRNAs are able to downregulate messenger levels as monitored by microarray experiments and (2) that these artificially downregulated mRNAs are usually underexpressed in the tissue where the miRNA is expressed. MiRNA may therefore induce a large-scale transcriptional shift towards a tissue-specific expression pattern. It is not clear yet whether messenger levels are reduced through a specific mechanism or as a consequence of translational repression, but this observation opens new avenues for monitoring and understanding miRNA-based gene regulation.

The 3′ UTR of eukaryotic transcripts, which hosts miRNA target sites, runs from the stop codon to the poly(A) site, where pre-mRNAs are cleaved and polyadenylated. In about half of human genes, several poly(A) sites are present, resulting in transcript isoforms with 3′ UTRs of different lengths produced from a single gene [9–11]. We questioned in this study whether expression levels would be affected when certain isoforms contain a microRNA target while others do not. Such a situation arises when a miRNA target is located downstream of the first poly(A) site, resulting in “long” isoforms containing the target and “short” target-free isoforms. Expressed sequence tag (EST) data are particularly well suited for such alternate transcript analysis, since a very large number of 3′ ESTs have been produced that extensively cover poly(A) site variations.

We carried out a systematic examination of 3′ UTRs containing multiple EST-supported poly(A) sites, looking for known miRNA targets and other phylogenetically conserved motifs. We grouped together genes containing an identical conserved motif located downstream of the first poly(A) site and, based on EST counts and EST library information, we assessed whether motif-containing and motif-free isoforms were differentially represented in specific tissues. We describe an application of this strategy to miR-1 and miR-124, the miRNAs first reported to cause tissue-specific transcript repression [5]. Encouraging results led us to apply the same principle on a larger scale. Analyzing the 312 highest-ranking motifs, we observed a significant enrichment in known miRNA targets and other regulatory elements, indicating that this principle may be exploited as a screen for motifs involved in transcript downregulation.

## Results

### Conserved Motifs and miRNA Targets in Alternatively Polyadenylated 3′ UTRs

In order to identify genes with alternative poly(A) sites, we mapped all 3′ ESTs and full-length cDNAs onto the human and mouse genome. After clustering EST/cDNA hits, we identified putative poly(A) sites based on several stringent criteria, including presence of at least three 3′ ESTs/cDNA from distinct libraries ending at each site, lack of potential internal priming, and presence of a poly(A) signal near the 3′ end of match. We then selected all human genes displaying two or more poly(A) sites in the 5-kb region downstream of their 3′-most annotated stop codon. This excluded most poly(A) variants resulting from splicing isoforms, but included putative poly(A) sites located downstream of current annotations. For each selected human gene, we obtained orthologs in mouse, rat, and dog from Ensembl [12], extracted the 5-kb genomic regions downstream of the stop codon in these genomes, and performed a multiple alignment of all four downstream regions. This produced 3,495 four-way alignments, hereafter termed “UTR alignments,” that were each truncated at the position of the 3′-most poly(A) site in human, resulting in an average alignment length of 2,197 nt.

Potential regulatory motifs were defined as a fully conserved 7-mer sequence in the four-species UTR alignment. Although we were potentially interested in any regulatory motif, this definition is in line with current models of miRNA targets [2,3,13]. We identified 373,948 (possibly overlapping) conserved 7-mers in 3,354 different UTR alignments (i.e., an average of 111 7-mers per UTR representing 14,640 distinct 7-mers out of 16,384 possible combinations). At this stage, conserved 7-mers may result from the presence of long conserved regions of unknown function in 3′ UTRs [14] as well as regulatory elements such as miRNA targets. Two-hundred and eleven known miRNAs have an exact Watson-Crick match of their 5′ seed (nt 1–7 or 2–8) to one of the conserved 7-mers. These potential miRNA targets are located in 2,017 distinct 3′ UTRs (i.e., about 60% of the gene set under study).

**Figure 1:**
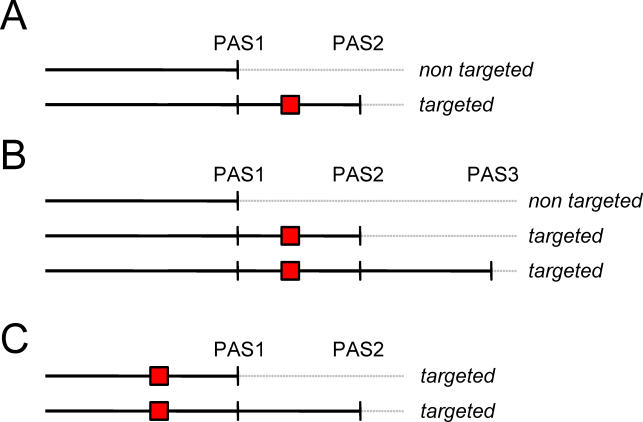
Possible Effects of Alternate Polyadenylation on 3′ UTRs Containing a Target Regulatory Motif (A) Two isoforms and motif located downstream of first poly(A) site: only long isoform is targeted. (B) Three isoforms and motif located between first and second poly(A) site: two longest isoforms are targeted. (C) Two isoforms and motif located upstream of first poly(A) site: all isoforms are targeted.

**Figure 2:**
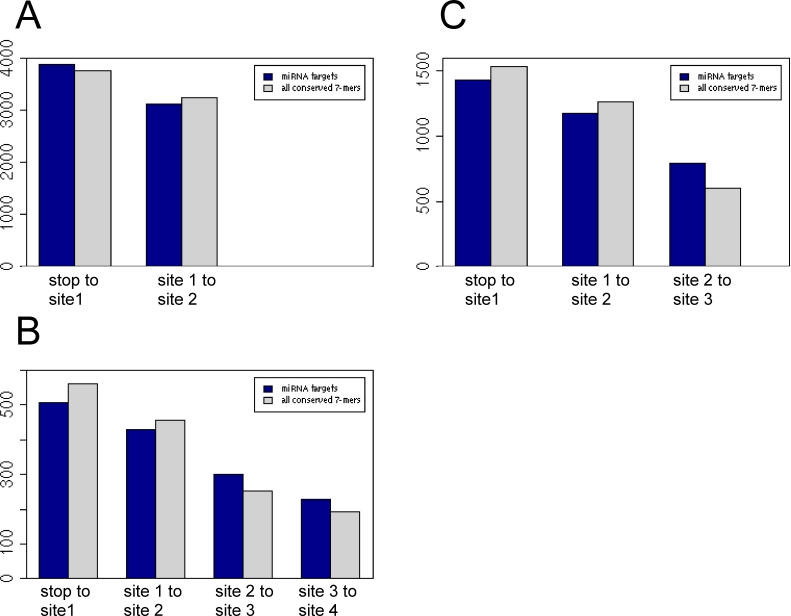
Distribution of Putative miRNA Targets and Other Conserved 7-mers in 3′ UTR Segments Delimited by Alternative Poly(A) Sites Number of putative miRNA targets is in dark blue, and total number of conserved 7-mers in gray, scaled down so that total 7-mers equals total miRNA targets. (A) Genes with two poly(A) sites. (B) Genes with three poly(A) sites. (C) Genes with four poly(A) sites.

Putative regulatory motifs located downstream of a poly(A) site were of particular interest to us since alternative usage of the poly(A) site should produce transcript isoforms that may or may not contain this motif, possibly leading to a differential regulation of isoforms (Figure 1A and 1B). For the sake of simplicity, such isoforms will be considered “targeted” or “nontargeted” and genes will be considered “differentially targeted” even though the “target” status of the conserved motif is not established yet. We first asked whether potential miRNA targets or other conserved motifs were seen to adopt a preferential location relative to alternative poly(A) sites. Figure 2 shows that there is no such preference. The numbers of potential miRNA targets and overall conserved 7-mers present in 3′ UTR sections delimited by 2, 3, or 4 poly(A) sites are shown. Numbers of conserved 7-mers generally decrease when considering more distal UTR sections. As consecutive sections have roughly the same average size, the density of conserved 7-mer decreases with distance from the stop codon. However, the distribution of putative miRNA targets does not differ significantly from that of other conserved 7-mers.

### Tissue-Specific Downregulation of Target-Containing Isoforms

Despite the previous observation, 52% of putative miRNA targets are located downstream of the first poly(A) site. Therefore, wherever a cognate miRNA is expressed, the resulting alternate transcripts may behave differently as a result of miRNA-mediated regulation. If certain miRNAs such as miR-1 and miR-124 are able to repress messenger levels, we expect a specific downregulation of targeted isoforms in tissues where such miRNAs are expressed. Taking advantage of the excellent EST coverage of human 3′ UTRs, involving thousands of different tissue-specific libraries, we set out to mine EST data for evidence of such regulations.

**Figure 3:**
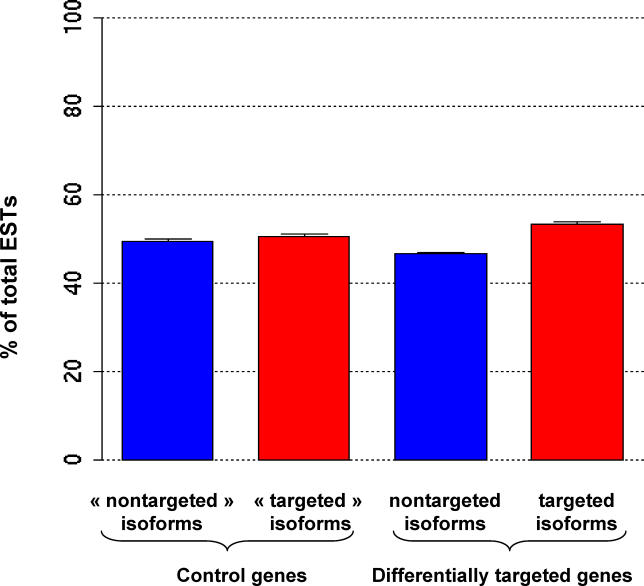
Relative EST-Based Expression Level of Target-Containing (Blue) and Target-Free (Red) Isoforms Total number of ESTs observed for each gene is scaled to 100. Left: control set contains isoforms from 1,875 alternatively polyadenylated genes, classified as “targeted” or “nontargeted” according to their location relative to a randomly selected position. Student paired test *p*-value for differential expression = 0.38. Right: test set contains isoforms from 562 genes containing targets for known miRNAs located downstream of first poly(A) site. Student paired test *p*-value for differential expression = 7.45 × 10^−5^.

The above-mentioned dataset contains 562 cases where alternate transcripts from the same gene are differentially targeted by a known miRNA (such as in Figure 1A or 1B, and excluding cases such as Figure 1C). Figure 3 presents average relative EST counts for targeted and nontargeted isoforms (i.e., the proportion of ESTs that correspond to the targeted isoform and the proportion corresponding to the nontargeted isoform). As a control set, we picked random sites in the 3′ UTRs of alternatively polyadenylated genes and selected those genes where the site felt downstream of the first polyA site. Average EST counts for such virtually targeted and nontargeted isoforms do not differ significantly (*p* = 0.38). On the other hand, the 562 genes that contain a potential miRNA target in their longer isoforms and not in their shorter isoform (Figure 3, right), display a moderate but significant overexpression of longer isoforms (*p* = 7 × 10^−5^), when all EST libraries are considered together.

**Figure 4:**
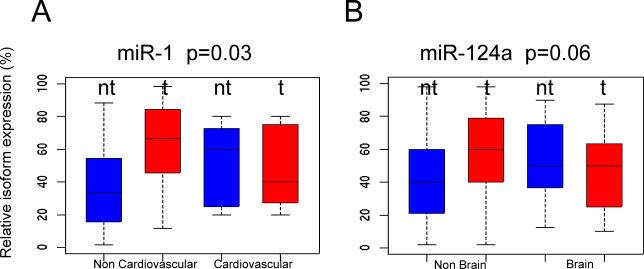
Relative EST-Based Expression of Isoforms Containing or Not Containing a Target for miR-1 or miR-124 “t” indicates targeted forms, “nt” indicates nontargeted forms. The *p*-value on top of each panel is the significance of the expression reduction of targeted forms between the two tissue contexts. (A) For genes targeted by miR-1, relative expression was measured based on EST libraries flagged as cardiovascular in eVOC (right, “cardiovascular”), and all other libraries (left, “noncardiovascular”). (B) For genes targeted by miR-124, relative expression was measured based on all EST libraries flagged as brain in eVOC (right, “brain”), and all other libraries (left, “nonbrain”).

For genes containing miR-1 or miR-124 targets, we expected that targeted isoforms would be downregulated in tissues where cognate microRNAs are known to be expressed. MiR-1 is preferentially expressed in heart and skeletal muscle, and miR-124 is preferentially expressed in brain [15,16]. Figure 4 shows the average relative EST-based expression levels of targeted and nontargeted isoforms in cardiovascular tissues for miR-1 and brain tissues for miR-124, compared to their relative expression in other tissues. While the level of targeted isoforms is usually higher than that of nontargeted isoforms in tissues taken as a whole, it is reduced in the tissue class where the cognate miRNA is expressed, with a one-way *T* test *p*-value of 0.03 for miR-1/cardiovascular, and 0.06 for miR-124/brain.

**Figure 5:**
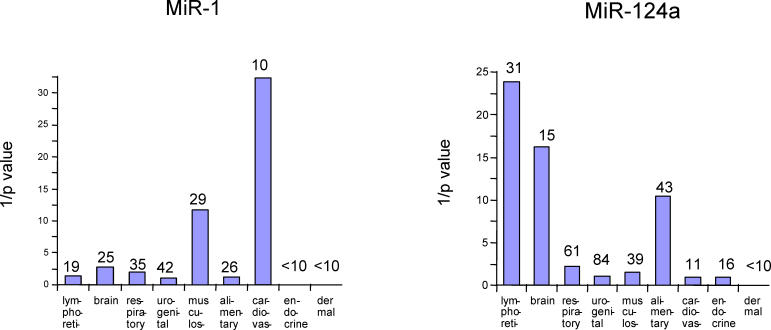
Inversed *p*-Value of Differential EST-Based Expression of Targeted Isoforms Between a Given Tissue Class and All Other Tissue Classes Combined (Student test, one-way) Numbers on top of bars indicate total numbers of targeted genes for which EST coverage was sufficient in this tissue class for both isoforms. No *p*-value was computed for tissue classes where less than ten genes were represented. Top-level tissue class “nervous” was replaced here by “brain” to avoid contamination by libraries from the peripheral nervous system.

Could this apparent specific repression of targeted isoforms be fortuitous? We repeated the analysis for other top-level tissue classes in the eVOC ontology, which describes tissue information in EST/cDNA libraries as a controlled set of terms. Figure 5 shows levels of repression of targeted versus nontargeted isoforms in each class, for genes containing miR-1 and miR-124 targets. We required that a given tissue class had EST coverage for at least ten differentially targeted genes to perform the analysis, which was not satisfied for all tissues. For miR-1–targeted isoforms, a stronger repression is observed in cardiovascular and musculoskeletal tissues, which agrees well with experimental data [15], even though repression in musculoskeletal tissue was not statistically significant (*p* = 0.09). For miR-124, the strongest repression is observed in lymphoreticular tissues, a class not reported to show miR-124 expression. However, brain tissues rank second in terms of differential isoform repression.

### Screening for miR Targets: The Disrep Procedure

**Figure 6:**
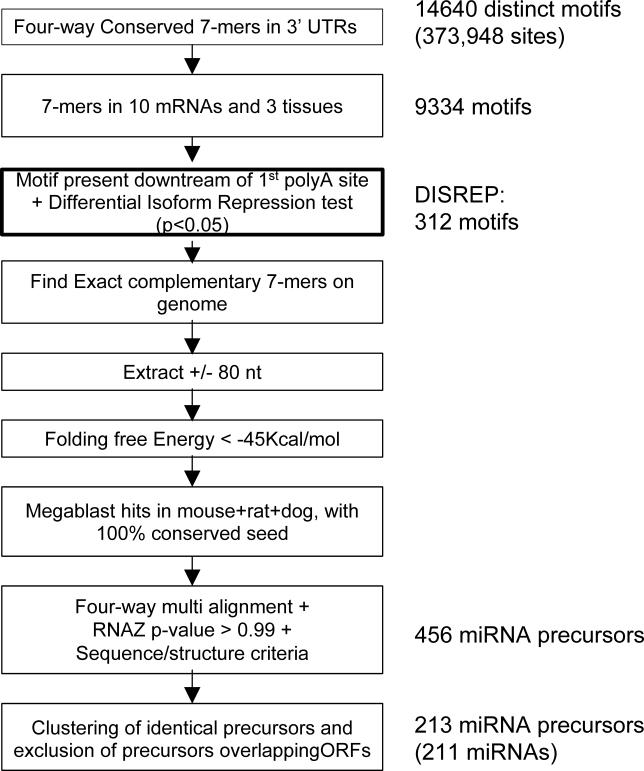
Overall Protocol for the Identification of Regulatory Targets and Subsequent Identification of Cognate miRNAs

These encouraging results prompted us to repeat this analysis for any conserved 7-mer sequence found 3′ of the first poly(A) site of a gene (Figure 6). From our initial set of 14,640 distinct conserved 7-mer motifs, we extracted those 9,334 motifs present in at least ten genes in each of three distinct tissue types (considering eVOC top-level tissue categories). Amongst these, 3,810 motifs were present downstream of the first poly(A) site. For each eVOC top-level tissue category, we measured average EST counts for targeted and nontargeted isoforms. A hit was recorded when the relative expression level of targeted forms in this tissue class differed significantly (one-way *T* test *p* < 0.05) from relative expression levels of targeted forms in all other tissues combined. This “differential isoform repression” (Disrep) procedure, combining the requirement for a motif to be present downstream of the first poly(A) site and the *p*-value criteria, identified 312 motifs associated with an apparent repression of targeted isoforms in one particular tissue class (Table S1).

While our initial working set of 9,334 motifs contained 260 targets for known miRNAs, 29 such targets were present in the final set of 312 motifs after application of the Disrep screen. This enrichment is highly significant (*p* = 1 × 10^−8^), especially when considering that all miRNAs may not necessarily disrupt transcript levels. Interestingly, other 3′ UTR regulatory motifs are overrepresented in the Disrep set (Table 1): destabilizing AU-rich elements (AREs) are enriched 5.2 times (*p* = 1.2 × 10^−4^) and Puf protein binding sequences that may be involved in enhancing mRNA decay [17] are enriched 15 times (*p* = 6.4 × 10^−3^).

**Table 1:**
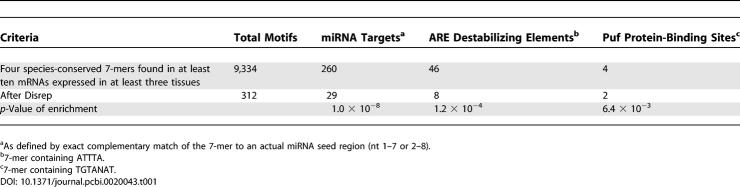
7-mer Motif Enrichment in miRNA Targets through Use of Disrep

**Figure 7:**
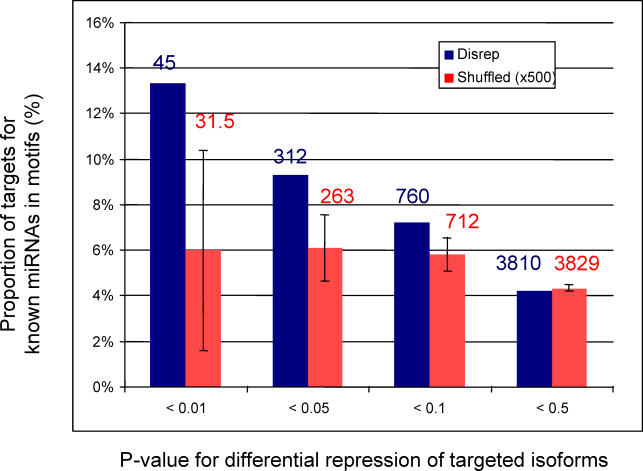
Proportions of Targets for Known miRNAs among Predictions Obtained at Different *p*-Value Ranges by the Disrep Procedure Numbers on top of bars give total sample size for this *p*-value range. Values are cumulative. Blue: test dataset; red: average of 500 shuffled controls (see text). Error bars provide standard deviation.

Our requirement for conserved motifs to be present in at least ten distinct mRNAs in each of three distinct tissues may also cause an enrichment in miRNA targets, independent of any differential repression. However, this constraint is applied prior to the Disrep screen (Figure 6) and therefore cannot account for the observed effect. The effectiveness of the Disrep screen in selecting true miRNA targets is supported by the inverse correlation between Disrep *p*-values and the proportion of targets for known miRNAs in the prediction set (Figure 7). This proportion increases continuously from 4% in low-scoring motifs to about 13% in high-scoring motifs.

As a control procedure, we randomly permuted the 9,334 conserved motifs identified prior to the Disrep procedure, in a manner that maintained the number of conserved 7-mers in each gene and the number of genes containing each 7-mer. We then applied the complete Disrep procedure (selection of motifs found downstream of the first poly(A) site and differential isoform repression test) using these permuted motifs. Motifs were sorted by Disrep *p*-value, and we measured the proportion of targets for true miRNAs in each *p*-value class. This whole control procedure was repeated 500 times, and average results are indicated by red bars in Figure 7. The enrichment in “true” targets with higher *p-*values is clearly absent in the control, which confirms that the Disrep screen alone causes the functional motif enrichment. None of the 500 control runs produced more predicted targets than observed in the test run at *p* = 0.05. However, absolute numbers of predictions in control runs (top of bars in Figure 7) reveal a relatively poor signal–noise ratio, ranging from 1.43:1 at *p* = 0.01 to 1.18:1 at *p* = 0.05. Much of this noise (i.e., low *p*-value motifs identified independently of the Disrep procedure) may result from bona fide miRNA targets, as prior constraints in our protocol (conserved motifs present in more than ten genes) are known to contribute to miRNA target identification. However, predictions that are specific to the Disrep screen are expected to represent only about 50 true biological elements in our set of 312 motifs.

### Differential Expression Measured from SAGE Data

To circumvent possible biases due to inaccuracies in EST counts, we undertook the same analysis using serial analysis of gene expression (SAGE) data to measure isoform expression level. Human SAGE sequences were mapped onto alternatively polyadenylated transcripts as described in Material and Methods . As there is no available eVOC mapping of SAGE libraries, we manually classified the 326 SAGE libraries into 27 different tissue types. After filtering out conserved 7-mers associated with less than ten genes and three different tissue types, we were left with 11,243 7-mers, containing 203 targets for known miRNAs. We submitted these 7-mers to the Disrep procedure, using the same parameters as with EST data, and 7-mers were ranked by *p*-value. Among the 1,001 motifs with a *p*-value lower than 0.01, 38 were targets of known miRNAs. This represents a highly significant enrichment (*p* = 7.8 × 10^−6^). All motifs with a SAGE-based Disrep *p*-value below 10 × 10^−3^ are presented in Table S2. As in the EST-based procedure, we observed an inverse correlation between SAGE-based Disrep *p*-values and the proportion of targets for known miRNAs among predictions (Figure S2). In addition, the enrichment in known miRNA targets in 1,000 shuffled control sets never attained the level observed in the nonshuffled set at *p*-values of 0.01 or 0.005. Finally, among the 312 highest-ranking motifs of the EST-based protocol, 33 were also found among the 312 highest *p*-values of the SAGE-based protocol. This enrichment is also highly significant (P = 3.2 × 10^−11^). The 33 motifs supported by both SAGE and EST data are shown in Table 2, along with *p*-values and tissue information. Unsurprisingly, tissue predictions from SAGE and EST data do not always coincide. Indeed, isoform repression may occur in different tissues for a single target, and all tissues are not equally sampled in EST and SAGE libraries. In any case, these combined results confirm the validity of the Disrep procedure independently of the type of transcript measure (EST or SAGE) used to monitor isoform expression.

**Table 2:**
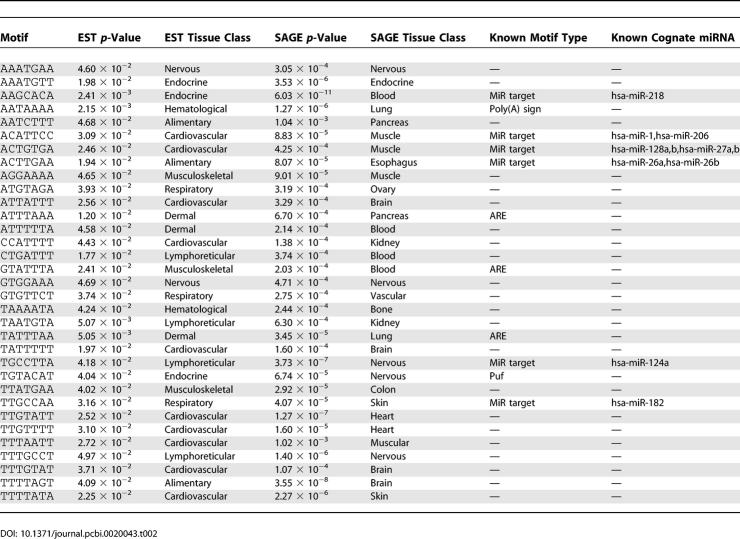
7-mer Motifs Associated to a Significant Disrep *p*-Value in Both the EST-Based and SAGE-Based Protocols

### Prediction of Cognate miRNAs

To complete this study by an experimentally testable set of predictions, we scanned the human genome for conserved miRNA precursors containing a “seed” sequence complementary to any of the 312 7-nt motifs. Our protocol (Figure 6) required (1) a perfect complementary 7-nt “seed” sequence; (2) a predicted folding free energy below −45 Kcal/mol in the 160-nt fragment around the seed; (3) a significant BLAST [18] match in the mouse, rat, and dog genomes; and (4) a hairpin-like secondary structure that was both correctly located relatively to the seed sequence and supported by the four genome sequences according to RNAz [19], a program that identifies optimal RNA structures in terms of conservation and folding free energy. Putative mature miRNAs were then derived from successful precursors by extending the seed sequence 13 nt to its 3′ end.

This procedure identified 456 potential human miRNA precursors, 417 of which did not overlap a known open reading frame (Table S3). After clustering overlapping precursors containing seeds that were separated by at most one nt, we obtained 213 distinct miRNA precursors and 211 distinct miRNAs (Table S4). The subset of 46 candidate miRNAs that would target 7-mer motifs supported by both EST and SAGE data is presented in Table 3. Of the final 211 precursors, 45 were present in the miRNA registry [20] and 38 more were predicted by recent computational studies [13,21,22], resulting in 128 novel candidates. Twenty-two of the 128 novel precursors match the opposite strand of known miRNA precursors and would thus involve transcription of minus strand in order to be expressed. About 9.5% of the 128 candidate miRNAs were located in the vicinity (<1 kb) of other known or predicted miRNAs, higher than the fraction of clustered miRNAs in predictions by Berezikov et al. (51/975 = 5.2%), but lower than that in the miRNA registry [20] (28% clustered). As expected, all 312 motifs did not meet a cognate miRNA: 43% remained orphan. A fraction of these orphan targets may result from an excessive stringency of our precursor identification protocol or may be recognized by factors other than miRNAs as in the case of Puf protein binding sites and AREs. A significant fraction of orphan targets may also be false positives. We thought that candidate targets containing the AAUAAA polyadenylation signal (five targets) could be such false positives, caused by the prevalence of this motif in 3′ UTRs. However, we found seven different candidate miRNAs matching these motifs, indicating that some miRNAs may target poly(A) signals.

**Table 3:**
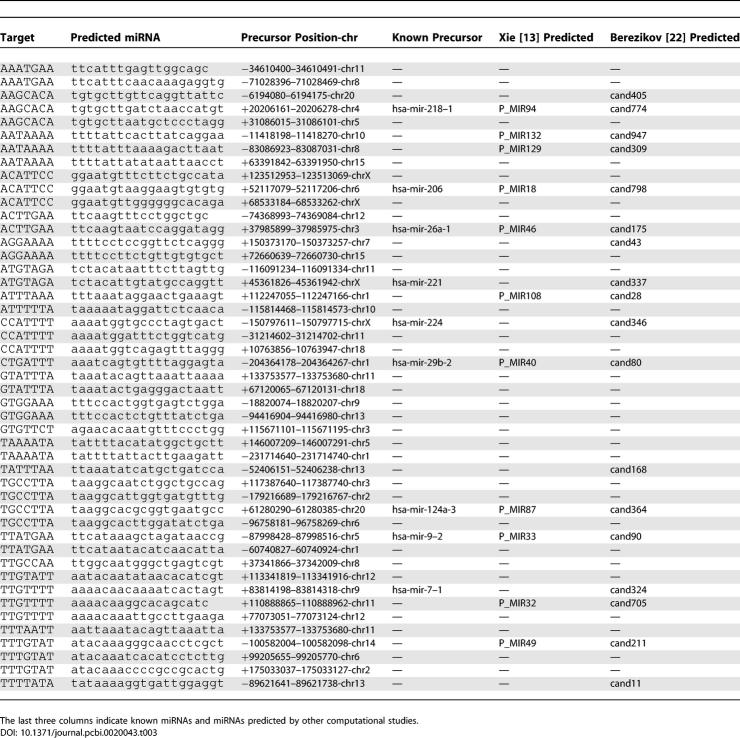
Predicted miRNAs Complementary to Targets Supported by Both EST and SAGE Data (Table 2)

## Discussion

At the outset of this study our intention was to observe the interplay of polyadenylation and miRNA targeting, two central mechanisms in the control of transcript fate. Our initial observation that transcript isoforms containing miRNA targets were generally not underexpressed compared to target-free isoforms (Figure 3) was at a first glance discouraging. Only when tissues known to express miR-1 and miR-124 were singled out did a tendency emerge for specific downregulation of isoforms containing targets for these miRNAs. Applying this analysis to other conserved motifs in 3′ UTRs, we extracted 312 motifs that may be associated to a differential repression of isoforms in specific tissues. This list is significantly enriched in true miRNA targets and other regulatory elements such as AREs or Puf-binding sequences; as a matter of fact, AREs can be considered miRNA targets, since these destabilizing elements were recently discovered to act through recognition by miR16 [23]. A significant background noise is observed, consisting of motifs identified without help of the Disrep procedure. Indeed, preliminary steps of our protocol involve extracting conserved 7-mers present in several different mRNAs and this constraint alone is known to select miRNA targets [13]. However, the quantity of additional motifs identified by the Disrep procedure cannot be accounted for by this effect, and the *p*-value–dependent enrichment in known regulatory targets indicates that differential repression of 3′ variants occurs and can be exploited to identify novel miRNA targets.

How significant, however, is the interplay of alternative polyadenylation and miRNA targeting in the overall process of posttranscriptional regulation? By regulating specific polyadenylation isoforms, miRNAs may up- or downregulate transcripts containing other regulatory elements. There are several known regulatory elements in animal 3′ UTRs, such as the iron response element, selenoprotein insertion sequence, or *Drosophila* translation control element, and it is likely that many remain to be identified. Knocking out transcript isoforms containing such elements could be an additional control lever for gene expression. Alternatively, this mechanism could simply provide a fine-tuning of gene expression by knocking down just part of the transcript population for a given gene. Admittedly, the latter seems like an unwieldy way to regulate messenger levels, involving synthesis of two or more isoforms and their subsequent tissue-specific degradation by microRNAs or other factors, although yet more “expansive” regulatory mechanisms have been observed.

A comparison of orthologous genes in human and mouse showed no specific conservation of the association between alternate polyadenylation and the presence of miRNA targets (Figure S1), suggesting the dual control of some genes by polyadenylation and miRNA targeting is more likely an accidental phenomenon than an essential physiological mechanism. Therefore, although miRNA targets can be under selection (hence conserved) in specific 3′ UTRs, the accidental occurrence of alternative polyA sites in these UTRs could produce isoforms escaping miRNA regulation without conferring a strong selective advantage or disadvantage. This chance event is a fortunate one, though, as it can be used as a tool for analyzing posttranscriptional regulation. The class of downregulatory motif identified here would not be easily detectable by monitoring genes as a single expression unit, using for instance microarray data, since transcriptional variations from one targeted gene to another would generally offset miRNA-based regulation. When comparing the expression of isoforms from the same gene, nontargeted isoforms act as naturally provided internal controls, allowing us to ignore transcriptional effects.

Most computational protocols for miRNA discovery to date have relied on seeking precursors through a combination of phylogenetic footprinting and free energy/sequence bias filters [22,24,25]. Recently, Xie et al. [13] have introduced a reverse approach in which putative targets are identified first, and cognate miRNA precursors are sought as phylogenetically conserved, complementary genomic sequences displaying an aptly folded structure. We used a similar “reverse” approach to identify potential miRNA partners for predicted motifs. However, where Xie et al. required conserved targets to occur in the order of 100 times in a genome, our target selection requires only ten target-containing UTRs, relying instead on differences in isoform expression. Due to these different selection criteria, our protocol is able to identify target (and hence miRNA) candidates that went unnoticed during previous scrutiny. Another important aspect of our procedure is its focus on motifs associated to transcript degradation or destabilization rather than translational repression. It now appears that a large fraction of animal miRNAs are able to reduce transcript levels [7,8] and therefore our procedure potentially identifies multiple miRNA targets. However, other types of regulatory motifs may also emerge. For instance, Zhang et al. have recently proposed that some 3′ UTR motifs may be controlling tissue-specific polyadenylation [26]. When favoring shorter isoforms, such an event could be detected by Disrep, although it is not a downregulation of longer isoforms, but instead an upregulation of shorter isoforms. Other classes of regulatory motifs that can be identified by our protocol include targets for unknown regulatory proteins or for novel types of antisense RNAs with a transcript repression effect. The latter is an exciting perspective that undoubtedly deserves attention.

## Materials and Methods

### Poly(A) site prediction.

3′ EST sequences from dbEST v. 01/06/05 and full-length cDNA sequences from H-Inv 1.8 [27] and FANTOM 2.01 [28] were cleaned for trailing poly(A) or poly(T) sequences and aligned to the repeat-masked human genome v.27.35a.1 and mouse genome v27.33c.1 using the Megablast program [18]. All hits presenting at least 95% identity with the genomic sequence were retained and clustered. Each cluster was analyzed using a sliding window to locate the most likely cleavage site, defined as the position where the window contains the most EST/cDNA ends. The following filters where then applied: (1) discard hits with more than 5 unmatched nt at cleavage site; (2) discard cleavage sites flanked by A-rich regions in the 50-nt downstream genomic sequence; and (3) retain only cleavage sites supported by at least 3 EST libraries and in which the 30-nt upstream genomic sequence contains any of the 11 variant poly(A) signals from Beaudoing et al. [9]. Selected poly(A) sites were then assigned to the nearest 5′ gene, provided that the 3′-most stop codon (Ensembl annotation [12]) for this gene was less than 5 kb from the poly(A) site. In order to favor tandem poly(A) sites over sites occurring in different splice variants, any internal site located upstream of the 3′-most stop codon was discarded.

### 3′ UTR alignments and conserved motifs.

Human transcripts with two or more predicted polyA sites and orthologs found in mouse, rat, and dog based on Ensembl Compara version 27_1 [13] were selected. For each ortholog group, we retrieved the longest human 3′ UTR sequence (from stop codon to the most distal poly(A) site, up to 5,000 nt) and UTRs from the other species, extended to 5 kb downstream of stop codon. Those orthologous 3′ UTRs were then anchored using Chaos [29] and aligned using Dialign [30]. Multiple alignments were truncated at the last position of the human sequence. 3′ UTR alignments were scanned for 7-nt motifs showing an exact four-way conservation and containing neither “N” nor gaps. All overlapping motifs were retained. We defined as putative miRNA targets those conserved motifs displaying an exact complementary match to the seed sequence (nt 1–7 or 2–8) of a known miRNA from the miRNA registry 6.0 [20].

### Expression levels of differentially targeted isoforms.

Differentially targeted isoforms were defined as follows: positions of all conserved motifs were determined relatively to alternative poly(A) sites for each gene. If a conserved motif was located upstream of the 5′-most poly(A) site as in Figure 1C, all isoforms were considered “targeted.” If the conserved motif was located between poly(A) sites *i* and *i* + 1 (Figure 1A or 1B), and absent upstream of site *i,* then the gene was considered “differentially targeted.” All isoforms ending at site *i* or shorter were considered “nontargeted,” while isoforms ending at site *i* + 1 or longer were considered “targeted.” For control purposes (Figure 3, left), positions were randomly picked in 3′ UTRs following the same site distribution as that of conserved targets for known miRNAs. Isoforms were then classified as “targeted” or “nontargeted” according to their location relative to this random point. Expression levels of isoforms were estimated based on EST counts, using the same ESTs used in poly(A) signal identification, thus ensuring that nonspecific ESTs compatible with two or more poly(A) isoforms were disregarded.

Tissue-specific expression was assessed using the eVOC 2.6 ontology description of expression states in EST libraries [31]. To avoid sampling issues, only top-level eVOC terms were considered, namely: alimentary system, cardiovascular system, dermal system, developmental anatomy, endocrine system, hematological system, lymphoreticular system, musculoskeletal system, nervous, respiratory system, unclassifiable, and urogenital system. For the analysis of miR-1 and miR-124 targets (Figure 5), tissue class “nervous” was replaced by lower-level class “brain.”

For studying the impact of a putative target on expression (Disrep procedure), each target was tested individually for a difference in the expression level of targeted isoforms of a given tissue type in comparison to the pooled expression level of all other targeted isoforms in other tested tissues. An eVOC tissue type was tested in relation to a given miRNA target only when at least ten differentially targeted genes were observed expressed in this tissue. Significant differences (paired one-way *t* test *p* < 0.05) allowed us to flag a predicted target as having a regulatory effect in a given tissue class.

### Expression levels computed from SAGE data.

We constructed putative 3′ UTR sequences by associating predicted polyA sites to the nearest Ensembl transcript and extracting the genomic sequence between the annotated stop codon and the cleavage site. This produced a total of 60,245 putative UTR sequences. SAGE data was downloaded from the National Center for Biotechnology Information Gene Expression Omnibus (NCBI GEO) server (http://www.ncbi.nlm.nih.gov/geo). These data include four platforms, GPL4, GPL1485, and GPL2750 for the NlaIII enzyme, and GPL6 for Sau3A, representing a total of 1,378,959 10-nt or 17-nt sequences from 326 distinct libraries. SAGE mapping to UTR sequences was performed in two stages. First, we looked for the 3′-most occurrence of a SAGE sequence in each UTR. Then we eliminated SAGE sequences that mapped to two different genes. Among the 60,245 putative UTR sequences, 35,091 were correctly associated to one SAGE sequence. We then manually parsed SAGE library annotations to classify expression information into 27 distinct anatomical categories (blood, bone, brain, breast, cartilage, cerebellum, colon, esophagus, eye, foreskin, heart, kidney, liver, lung, muscle, nervous system, ovary, pancreas, peritoneum, placenta, prostate, skin, spinal cord, stem cells, stomach, thyroid, and vascular). The SAGE-based Disrep procedure used this library information and SAGE counts per library in the same way as above for EST eVOC libraries and EST counts.

### Identification of cognate miRNAs.

We searched the human genome (Ensembl human version 27_35a) for reverse-complements of the selected 7-mers motifs and extracted the −80/+80-nt region around this “seed” sequence. Regions with an RNAfold [32] folding energy ≤ −45 Kcal/mol were retained as queries for a Megablast search (E-value cutoff ≤ 1 × 10^−5^, Word size = 16) of the mouse (Ensembl mouse version 27_33c), rat (Ensembl rat version 27_3e), and dog (Ensembl dog version 27_1) genomes. Human sequences with hits in all three species, including a fully conserved reverse-complement motif, were retained. Four-way alignments of human sequences and their highest scoring hits were performed with ClustalW [33]. We retained those alignments with a RNAz [19] RNA-class *p*-value ≥ 0.99 and at least 95% identity in the putative mature miRNA region (7-mer + 13 nt on 3′). Each human sequence was further folded with RNAfold [32] using the consensus secondary structure as a constraint to produce a human-specific structure, as suggested by Gardner and Giegerich [34]. The folds thus obtained were filtered in regards to further structural criteria: presence of a unique hairpin loop, minimum of 20 bp, putative miRNA region not overlapping with the apical loop, no bulge longer than 4 nt, and no more than 6 bp mismatches. Overlapping precursors containing seed sequences separated by at most 1 nt were clustered. Predicted precursors that overlapped a known translated exon by at least 1 nt were removed.

## Supporting Information

Figure S1 Topology of Mouse Orthologs of Human Genes with Differentially Targeted Isoforms (i.e., Containing a Conserved 7-mer Motif [red box] Downstream of First poly(A) Site) Three topologies are possible for the orthologous mouse genes: (A) no alternative polyadenylation; (B) alternative polyadenylation with motif located downstream of first poly(A) site; and (C) alternative polyadenylation with motif located upstream of first poly(A) site. Number of observations in each class are given for known miRNA targets and for other conserved 7-mers.
(20 KB PPT)

Figure S2 Results of SAGE-Based Disrep Blue bars show the proportion of targets for known miRNAs among predictions obtained at different Disrep *p*-value ranges. Here, the Disrep procedure uses SAGE data to measure isoform expression levels. Numbers on top of bars give total sample size for this *p*-value range. Values are cumulative. Red bars present proportions of targets for known miRNAs obtained after motif randomization, as follows. We randomly distributed the 11,243 7-mer motifs into five bins of same size as obtained in the test run (200 in the first bin, 801 in the second, 1,451 in the third, 1,154 in the fourth, and 7,525 in the last) regardless of their *p-*values. Proportions of targets for known miRNAs in each bin were computed for 1,000 such shuffled sets and averaged. Error bars provide standard deviation.
(29 KB PPT)

Table S1 Putative Regulatory 7-nt Motifs Identified by the Disrep Procedure Using EST Data All 312 motifs with Disrep *p* < 0.05 are shown.
(56 KB XLS)

Table S2 Putative Regulatory 7-nt Motifs Identified by the Disrep Procedure Using SAGE Data All 276 motifs with Disrep *p* < 0.001 are shown.
(54 KB XLS)

Table S3 Putative Human miRNA Precursors Containing a Seed Sequence Complementary to Disrep Targets (121 KB XLS)

Table S4 Putative Human miRNA Precursors Containing a Seed Sequence Complementary to Disrep Targets, after Clustering of Overlapping Precursors (78 KB XLS)

## Abbreviations

ARE: AU-rich element
Disrep: differential isoform repression
EST: expressed sequence tag
miRNA: microRNA
SAGE: serial analysis of gene expression
UTR: untranslated region
